# Transcriptomic and Proteomic Analysis of the Skeletal Muscle Revealed the Effects and Mechanism of Mulberry Leaf Flavonoids on Alleviating Exercise-Induced Muscle Damage in Mongolian Horses

**DOI:** 10.3390/ani16101548

**Published:** 2026-05-18

**Authors:** Aopan Geng, Xuejiao Wang, Lianhao Li, Sarah Cowie, Dongyi Bai, Manglai Dugarjaviin, Xinzhuang Zhang

**Affiliations:** 1 Inner Mongolia Key Laboratory of Equine Science Research and Technology Innovation, College of Animal Science and Technology, Inner Mongolia Agricultural University, Hohhot 010018, China; gengaopan@163.com (A.G.);; 2 Rural Revitalization Research Institute, Inner Mongolia Agricultural University, Hohhot 010018, China; wangxuejiao881019@163.com; 3 School of Psychology, The University of Auckland City Campus, Private Bag 92019, Auckland 1142, New Zealand

**Keywords:** Mongolian horses, mulberry leaf flavonoids, exercise-induced muscle damage, transcriptomic, proteomic

## Abstract

The scientific evidence regarding the use of plant-derived extracts to alleviate exercise-induced muscle damage in horses remains limited. Mulberry leaf flavonoids (MLFs) are the primary bioactive constituents of a traditional medicinal plant and are potent antioxidants. The aim of this study was to investigate the protective effects of MLFs against exercise-induced muscle damage. In this study, twelve Mongolian horses were used in a 3 × 3 Latin square design to investigate the protective effects of MLFs. Our results showed that high-intensity exercise negatively impacted the immune status, metabolic state, myofibrillar structure, and antioxidant capacity of the horses. Conversely, MLFs significantly reduced blood levels of white blood cells (WBC), monocytes (MON), aspartate aminotransferase (AST), creatine kinase (CK), and malondialdehyde (MDA) across various exercise distances and during recovery. Simultaneously, MLFs increased serum glutathione peroxidase (GPx), superoxide dismutase (SOD), and total antioxidant capacity (T-AOC). Mechanistically, transcriptomic analysis revealed that dietary MLFs upregulated genes associated with myofibrillar structural proteins (*MYOZ2*, *MYOM3*), the antioxidant defense system (*GPX3*, *SOD3*), and skeletal muscle satellite cell proliferation and differentiation (*MYOD1*, *MRF6*). Furthermore, quantitative proteomics indicated the enrichment of the PI3K-Akt and TGF-β signaling pathways, as well as ECM–receptor interactions, suggesting their potential involvement in regulating protein metabolism and facilitating myofibrillar restoration. Overall, MLFs effectively alleviated inflammation, metabolic disorder, and exercise-induced muscle damage. Under the tested conditions, a daily dosage of 10 g MLFs provided superior protective effects.

## Simple Summary

Prolonged high-intensity training in racehorses induces severe oxidative stress and muscle damage. Dietary supplementation with mulberry leaf extract effectively ameliorates these conditions, as evidenced by reduced blood biomarkers for inflammation and muscle injury. Furthermore, transcriptomic and proteomic analyses of muscle tissue demonstrated significant enrichment in specific cellular signaling pathways, suggesting that the extract potentially modulates protein metabolism and facilitating the restoration of damaged muscle fibers. These findings clarify the molecular mechanisms behind the antioxidant properties of mulberry extract and provide a clearer understanding of its potential as a targeted nutritional intervention for exercise-induced muscle damage.

## 1. Introduction

Horse racing is an important economic industry in at least 71 countries worldwide, including developed nations such as the United States, Canada, and Japan [1]. This industry relies heavily on horses’ capacity for high-intensity exercise, while prolonged or intense exertion readily predisposes them to muscular injury, known as exercise-induced muscle damage (EIMD) [2]. Clinically, EIMD commonly presents with symptoms including impaired muscle force-producing capacity, decreased range of motion [3], delayed onset muscle soreness [4], and swelling of the affected limb. More specifically, the hallmark of EIMD involves two progressive injury processes: (1) primary muscle damage caused by repetition of non-uniform sarcomere lengthening and mechanical stress on a smaller number of muscle fibers, and (2) secondary muscle damage arising from calcium regulatory system disruption [5]. This calcium homeostasis imbalance further exacerbates tissue damage via inflammatory responses [6,7]. Simultaneously, high-intensity exercise enhances mitochondrial respiration to meet muscle oxygen and energy requirements, which in turn generates reactive oxygen species (ROS) including superoxide anions (O_2_•^−^), hydroxyl radicals (•OH), and hydrogen peroxide (H_2_O_2_) [8]. When the endogenous antioxidant defense mechanisms of the cell are overwhelmed, as occurs during excessive production of these ROS, horses may suffer from oxidative stress damage. This risk is not limited to specific horse types: endurance horses [9], show jumpers [10], and thoroughbred racehorses [11] are susceptible to oxidative stress during moderate- to high-intensity exercise [12]. Importantly, high ROS levels directly impair the muscle fiber membrane and mitochondria, leading to skeletal muscle contraction dysfunction and inducing muscle fatigue [13]. Oxidative stress is closely linked to most chronic inflammatory diseases [14].

Exogenous antioxidant supplements such as plant extracts [15] and coenzyme Q10 [16] are widely used in horses, and other supplements such as taurine [17] are widely used in humans to prevent or reduce exercise-induced muscle damage, spurring considerable research into their potential benefits. However, despite being a common practice among horse owners, trainers, veterinarians, and breeders, the application of plant extracts as exogenous antioxidant supplements to reduce exercise-induced oxidative damage in horses lacks peer-reviewed scientific evidence [15]. Information on their effects is primarily based on personal experiences rather than rigorous scientific research. Recent studies have explored the use of *Silybum marianum* and a combination of *Verbascum thapsus*, *Curcuma longa*, and *Boswellia serrata* in sport horses [18,19]. These plants display anti-inflammatory and antioxidant properties in vitro. However, no enhancement in antioxidant defenses was observed in these studies, as evidenced by the measurements of serum T-AOC, GPx activity, and the mRNA expression levels of *NFE2L2* and *SOD*. Thus, further rigorous scientific research is needed to substantiate the efficacy of plant extracts as antioxidant supplements for enhancing equine performance and animal welfare.

Mulberry leaf serves not only as a traditional medicinal plant but also as a high-quality livestock forage. It is particularly rich in bioactive flavonoids—including morachalcone D, rutin, mulberroside, quercetin, kaempferol, and astragaloside [20] which demonstrate significant antioxidant and anti-inflammatory properties. Mulberry leaf flavonoids (MLFs) have been shown to activate the Nrf2 signaling pathway and enhance antioxidant defenses [21]. Moreover, chronic intake of flavonoids can stimulate mitochondrial biogenesis, thereby enhancing muscle energy supply and endurance [22]. Our in vitro studies showed that MLFs enhanced antioxidant enzyme activity, improved mitochondrial respiration, and boosted cellular energy metabolism effectively mitigating H_2_O_2_-induced oxidative damage in equine skeletal muscle satellite cells via the Nrf2 signaling pathway [23]. It should be noted that while MLFs show promise in vitro, their actual efficacy in vivo remains to be validated due to the potential low bioavailability and complex metabolism of flavonoids in the gastrointestinal tract. Based on these findings, we hypothesize that MLFs can mitigate muscle damage in horses during high-intensity exercise. Therefore, the aim of this study was to evaluate the protective effects of dietary MLF supplementation on exercise-induced muscle damage in Mongolian horses, and to elucidate the underlying molecular mechanisms through integrated transcriptomic and proteomic analysis.

## 2. Materials and Methods

### 2.1. Animals Management

Twelve healthy Mongolian stallions (aged 24 ± 1.5 months) with similar genetic backgrounds and body conformation were selected from Xilin Gol League, Inner Mongolia Autonomous Region. All horses were grazed on the same natural pasture for standardized adaptive training and confirmed to be free of clinical disease through baseline hematological screenings. Twelve horses were randomly assigned to three dietary treatments under free-grazing conditions: a control group (NC, 0 g/d) and two MLF-supplemented groups (Low, 5 g/d; High, 10 g/d) (Table S1). The MLFs (purity = 50%, solid powder) were manufactured by Shaanxi Sinuote Biotech Co., Ltd. (Xi’an, China), utilizing the extraction conditions previously optimized in our laboratory via response surface methodology (55% ethanol, 1:30 solid-liquid ratio, at 50 °C for 17 min). The primary chemical compositions of the MLFs were previously identified in our metabolomics study [23]. The daily MLFs dosage was dissolved in water and administered via oral drenching using a dosing syringe before morning grazing to ensure complete intake. Synchronously, the control group (0 g/d) was administered an equal volume of physiological saline via oral drenching.

### 2.2. Study Design

Using a replicated 3 × 3 Latin square design consisting of three 20 d periods separated by 15 d washout intervals to ensure metabolic clearance. At the end of each period, a 20 km high-intensity exercise test was conducted on a closed, fixed track (5 km per round trip) in Xilin Gol League, Inner Mongolia (altitude: 1377 ± 5 m). During the test, the horses were ridden by experienced riders at an average target speed of 14.6 km/h to ensure a high-intensity workload. Heart rate (HR) and running speed were monitored in real-time using a Polar Vantage VV watch paired with an H10 heart rate sensor (Polar Electro Oy, Kempele, Finland). Average heart rate and running speed did not differ significantly among the three treatments during the exercise test (*p* > 0.05, Table S2).

### 2.3. Sample Collection

Blood samples were collected via jugular venipuncture before (0 km), during (at 5 km and 20 km), and after (1 and 4 h post exercise). One blood sample was collected into EDTA-K2 anticoagulant tubes (Kangweishi, Shijiazhuang, China) for hematological analysis, and the other was centrifuged at low speed to collect the supernatant, which was stored at −80 °C for serum biochemical and antioxidant assays. Following the third 20 d feeding phase, muscle biopsies were performed at two time points: immediately after the 20 km exercise and again 24 h later. For each procedure, horses were sedated (butorphanol and detomidine, 0.02 mg/kg BW each), and samples were extracted from the gluteus medius muscle (5 cm) using a Bergström needle [24]. A 1 mm^3^ portion was fixed for transmission electron microscopy (TEM), while the remainder was snap-frozen in liquid nitrogen and stored at −80 °C for antioxidant and multiomics analyses.

### 2.4. Blood Parameters

Hematological parameters (WBC, Lymph, Mon, Gran, RBC, HGB) were analyzed using an automated analyzer (BC-2800VET, Mindray, Shenzhen, China). Serum biochemical parameters (LDH, ALP, AST, UREA, CREA, TC, TG, GLU, CK) were measured using a BS-180VET analyzer (Mindray, Shenzhen, China). Serum and muscle antioxidant markers (GPx, SOD, CAT, T-AOC, MDA) were assessed using commercial kits (Nanjing Jiancheng Bioengineering Institute) following the manufacturer’s protocols.

### 2.5. TEM Analyses

The 1 mm^3^ gluteus medius samples were fixed in 2.5% glutaraldehyde and 2% paraformaldehyde at 4 °C. After washes in 0.1 M phosphate buffer, tissues were fixed with 1% osmium tetroxide and 1.5% potassium ferrocyanide, stained with 1% uranyl acetate, dehydrated in an ethanol gradient, and embedded in epoxy resin. Ultrathin sections (80 nm) were cut using an ultramicrotome (EM UC7, Leica, Microsystems, Vienna, Austria), double-stained with uranyl acetate and lead citrate, and observed under a TEM (H-7650B, Hitachi, Tokyo, Japan).

### 2.6. Transcriptomic and Proteomic Analyses

Samples of gluteus medius muscle collected immediately after exercise and at 24 h post-exercise from horses in the NC (n = 3) and High (n = 4) groups were subjected to transcriptomic and proteomic sequencing (Shanghai Majorbio Biopharmaceutical Technology Co., Ltd., Shanghai, China). One horse in the NC group was excluded from the omics analysis due to the failure of obtaining a sufficient muscle biopsy sample. Briefly, for the transcriptomic workflow, total RNA was extracted from gluteus medius tissue and placed into RNase free centrifuge tubes. RNA quality was assessed using the Agilent 5300 Bioanalyzer (Agilent Technologies, Santa Clara, CA, USA). Qualified RNA samples underwent mRNA purification and fragmentation. cDNA synthesis was performed using PrimeScript™ RT Master Mix (catalog RR036A, Takara Bio, Kusatsu, Japan) to reverse transcribe the RNA. Library construction was carried out on an Illumina platform using the NovaSeq Reagent Kit (Illumina, San Diego, CA, USA), followed by sequencing of muscle-derived mRNA. Sequencing reads were trimmed and quality controlled with fastp software (https://github.com/OpenGene/fastp, accessed on 6 October 2025). Subsequently, the filtered clean reads were aligned to the *Equus caballus* reference genome using HISAT2 (http://ccb.jhu.edu/software/hisat2/index.shtml, accessed on 6 October 2025). Expression levels of genes and transcripts were quantified with RSEM software (http://deweylab.github.io/RSEM/, accessed on 6 October 2025). Gene abundance was quantified. Differential expression analysis was conducted using DESeq2 (http://bioconductor.org/packages/stats/bioc/DESeq2, accessed on 6 October 2025). Genes with |log_2_ fold change| > 2 and adjusted *p* ≤ 0.05 were defined as significantly differentially expressed genes (DEGs).

For the proteomic analysis: 100 mg of gluteus medius tissue was homogenized in lysis buffer (8 M urea and 1% SDS) containing PMSF, centrifuged at 12,000× *g* for 2 min, and the supernatant was quantified using a BCA assay. A total of 100 µg of quantified protein was brought to 90 µL with lysis buffer, reduced with TCEP at 37 °C for 60 min, alkylated with iodoacetamide in the dark at room temperature for 40 min, precipitated with acetone, resolubilized in 50 mmol/L TEAB, and digested overnight at 37 °C with trypsin at a 1:50 enzyme to protein ratios. The resulting peptides were labeled at room temperature with TMT reagents (incubated with acetonitrile for 2 h, reaction quenched with hydroxylamine), pooled in equal amounts, and vacuum dried. The peptide samples were first subjected to high pH reversed-phase liquid chromatography fractionation on an ACQUITY UPLC BEH C18 Column (1.7 µm, 2.1 mm × 150 mm, Waters, Milford, MA, USA) at room temperature, with a flow rate of 200 μL/min and a 48 min gradient elution. The collected fractions were combined and vacuum-concentrated, then subjected to nano LC-MS/MS analysis on a C18 column (75 μm × 25 cm, Thermo Fisher Scientific, Waltham, MA, USA) at room temperature (Easy-nLC 1200 coupled with Q Exactive HF-X mass spectrometer, Thermo Fisher Scientific, Waltham, MA, USA). The raw mass-spectrometry files were processed with a pre-established *Equus caballus* protein database (e.g., UniProt, NCBI protein libraries, or custom genome/transcriptome-specific databases) for search and analysis. Proteins showing an absolute expression fold change ≥1.2 and adjusted *p* ≤ 0.05 were defined as differentially expressed proteins (DEPs). KEGG pathway enrichment for DEGs and DEPs was performed, with Padjust < 0.05 indicating significant enrichment. All data analyses were conducted on the Majorbio Cloud Platform (www.majorbio.com). Differential expression analysis in the transcriptomic and proteomic data was conducted using two-tailed *t*-tests, with Benjamini–Hochberg *p*-value corrections applied to control the false discovery rate. KEGG enrichment for DEGs and DEPs was conducted using the clusterProfiler R package on the Majorbio Cloud Platform, utilizing a hypergeometric test to determine statistically significant pathway enrichment (adjusted *p* < 0.05).

### 2.7. Quantitative Real-Time PCR

The extraction of RNA from muscle tissue, followed by reverse transcription and quantitative PCR, was conducted using established methods [25]. Trizol reagent (Thermo Fisher Scientific, Inc., Boston, MA, USA) was employed to isolate total RNA from the muscle samples, which was then converted into cDNA with a reverse transcription kit (Takara Biotechnology Co., Ltd., Dalian, China). The primer sequences used are listed in Table S3. cDNA amplification was carried out using a CFX 96 Real-Time PCR Detection System (Bio-Rad, Hercules, CA, USA), with GAPDH serving as the internal control gene. The relative mRNA expression levels of the target genes were determined using the 2^−ΔΔCt^ method.

### 2.8. Statistical Analysis

Hematological, biochemical, and antioxidant parameters were analyzed using the Linear Mixed Model (LMM) procedure in IBM SPSS Statistics 26.0 with a sample size of 12 horses. To correctly evaluate this crossover-style replicated Latin square design with repeated measures, the LMM included MLF dosage (M: 0, 5, or 10 g/d), recovery time (T: 0, 1, 4, 24 h) or exercise distance (D: 0, 5, and 20 km) and their interactions as fixed main effects, while the individual horse was modeled as a random effect. The normality of the standardized residuals was verified using Shapiro–Wilk tests. In the tabulated results, *P*_M_ denotes the main effect of dietary MLFs supplementation, *P*_D_ denotes the main effect of exercise distance, and *P*_T_ denotes the main effect of post-exercise recovery time. The interaction effects between exercise distance and MLFs dosage and between rest time and MLFs dosage are represented by *P*_D×M_ and *P*_T×M_, respectively. A Compound Symmetry covariance structure was applied to account for repeated measurements on the same animal. Estimated Marginal Means and corresponding Pooled Standard Errors Mean (SEM) were calculated, with post hoc pairwise comparisons performed using the Least Significant Difference test. Statistical significance was defined as *p* < 0.05.

## 3. Results

### 3.1. Hematological Parameters

All experimental groups maintained hematological parameters within normal reference ranges during both the exercise and post-exercise recovery periods (Table 1 and Table 2). Notably, there were no significant interaction effects between MLFs dosage and either exercise distance (*P*_D×M_ > 0.05) or recovery time (*P*_T×M_ > 0.05). Consequently, the main effects of dietary MLF supplementation and physical activity/rest were evaluated independently.

For leukocyte subpopulations (WBC, Mon, Gran, and Lymph), WBC, Mon, and Gran counts were lower in the MLF-supplemented groups in a dose-dependent manner, with the 10 g/d group maintaining the lowest levels compared to the 0 g/d and 5 g/d groups throughout both exercise and recovery (*P*_M_ < 0.05). The main effect of MLFs on Lymph counts showed a trend toward reduction during exercise (*P*_M_ = 0.090) but was not significant during recovery (*P*_M_ = 0.426). During the exercise phase, these immune cells increased progressively from 0 to 20 km (*P*_D_ < 0.05), but progressively decreased from 0 h to 4 h during the post-exercise recovery period (*P*_T_ < 0.001). Regarding erythrocytic indices (RBC and HGB), levels were significantly higher in both the 5 g/d and 10 g/d MLF-supplemented groups across both phases (*P*_M_ < 0.05), but also varied significantly with exercise distance, generally peaking at the 5 km mark (*P*_D_ < 0.05). However, during the recovery phase, they remained relatively stable and were unaffected by rest time (*P*_T_ > 0.05).

### 3.2. Biochemical Parameters

As presented in Table 3 and Table 4, there were no significant interaction effects between MLFs dosage and either exercise distance (*P*_D×M_ > 0.05) or post-exercise recovery time (*P*_T×M_ > 0.05) for any of the measured serum biochemical parameters. Therefore, the main effects of dietary treatment and exercise/recovery were analyzed independently.

The serum activities of AST and CK were dose-dependently lower in the MLF-supplemented groups throughout both the exercise and recovery phases (*P*_M_ < 0.001), with the 10 g/d group consistently exhibiting the lowest concentrations. Similarly, LDH levels were lower in the MLF-supplemented groups during exercise (*P*_M_ = 0.041) and showed a trend toward reduction during recovery (*P*_M_ = 0.056). ALP activity was significantly higher in the MLF-supplemented groups across both phases (*P*_M_ < 0.001).

The concentrations of UREA, CREA, and TC were significantly lower in the MLF-supplemented groups across both experimental phases (*P*_M_ < 0.05). TG levels were significantly lower in the MLF-supplemented groups during exercise (*P*_M_ = 0.039) but did not differ significantly among groups during recovery (*P*_M_ = 0.322). GLU remained unaffected by dietary treatments in both phases (*P*_M_ > 0.05). During the exercise challenge, almost all monitored biochemical parameters, including AST, CK, UREA, CREA, TC, TG, and GLU, increased progressively from 0 to 20 km (*P*_D_ < 0.05). Conversely, during the post-exercise recovery period, the serum concentrations of these same parameters progressively declined from 0 h to 4 h (*P*_T_ < 0.05). Meanwhile, LDH remained relatively stable during rest (*P*_T_ = 0.978), whereas ALP activity continued to increase (*P*_T_ = 0.001).

### 3.3. Antioxidant Indices in Serum

As shown in Table 5 and Table 6, no significant interaction effects were observed between MLFs dosage and either exercise distance (*P*_D×M_ > 0.05) or post-exercise recovery time (*P*_T×M_ > 0.05) for any serum antioxidant indices. Consequently, the main effects of dietary treatment and exercise/recovery were analyzed independently.

T-AOC, SOD, and GPx activities were higher in the MLF-supplemented groups in a dose-dependent manner across both the exercise and recovery phases, with the 10 g/d group maintaining the highest levels compared to the 0 g/d and 5 g/d groups (*P*_M_ < 0.05). Conversely, serum MDA concentrations were dose-dependently lower in the MLF-supplemented groups in both phases, with the 10 g/d group exhibiting the lowest levels (*P*_M_ < 0.001). The main effect of MLFs on CAT activity was not significant in either experimental phase (*P*_M_ > 0.05). During the exercise phase, the serum activities of T-AOC, SOD, CAT, and GPx progressively decreased from 0 to 20 km, while MDA concentrations progressively increased (*P*_D_ < 0.05). Conversely, during the post-exercise recovery period, T-AOC, SOD, and GPx activities progressively increased from 0 h to 4 h (*P*_T_ < 0.05), accompanied by a progressive decrease in MDA concentrations (*P*_T_ = 0.001). Meanwhile, CAT activity remained stable during the recovery phase and was unaffected by rest time (*P*_T_ = 0.131).

### 3.4. Antioxidant Indices in Muscle

As presented in Table 7, no significant interaction effects were observed between MLFs dosage and post-exercise recovery time for any of the measured muscle antioxidant indices (*P*_T×M_ > 0.05). Consequently, the main effects of dietary treatment and recovery time were evaluated independently. The muscle activities of T-AOC, SOD, CAT, GPx, and the concentration of MDA did not differ significantly between the 10 g/d MLFs group and the 0 g/d group (*P*_M_ > 0.05). Muscle T-AOC levels exhibited a progressive increase from 0 h to 24 h post-exercise (*P*_T_ = 0.02). However, the muscle activities of SOD, CAT, and GPx, as well as MDA concentrations, remained statistically unchanged during this 24 h recovery window (*P*_T_ > 0.05).

### 3.5. Myofibrillar Structure

**Figure 1:**
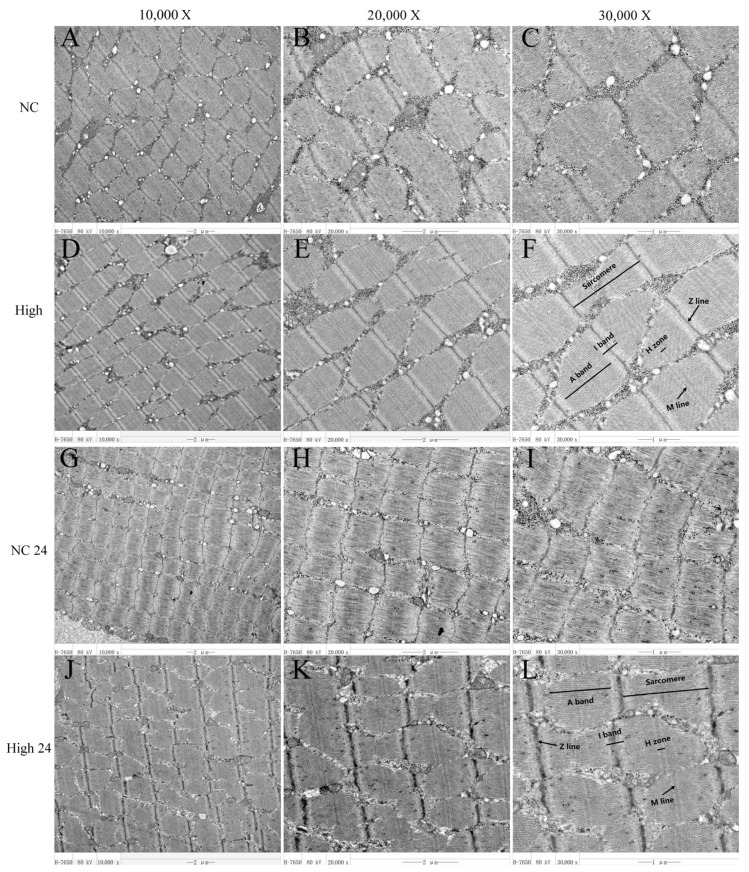
Effects of MLFs on myofibrillar structure in Mongolian horses at 0 h and 24 h post-exercise. (**A**–**C**) NC treatment: 10,000×, 20,000× and 30,000×. (**D**–**F**) High treatment: 10,000×, 20,000× and 30,000×. (**G**–**I**) NC 24 treatment: 10,000×, 20,000× and 30,000×. (**J**–**L**) High 24 treatment: 10,000×, 20,000× and 30,000×.

Qualitative morphological evaluation of the gluteus medius muscle via TEM is presented in Figure 1. Immediately following the 20 km exercise (0 h post-exercise), the myofibrillar architecture in the NC group exhibited localized disorganization, characterized by irregular Z-line alignment and less distinct sarcomere banding (Figure 1A–C). In contrast, the High-MLFs group (10 g/d) displayed distinctly aligned Z-lines, intact myofilaments, and clearly identifiable A-bands, I-bands, H-zones, and M-lines (Figure 1D–F).

At 24 h post-exercise, residual structural irregularities, including slight focal misalignment of Z-lines, remained visible in the NC group (Figure 1G–I). Conversely, the High-MLFs group maintained a highly organized and tightly packed sarcomere structure, demonstrating continuous, straight Z-lines and distinct cross-striations across the observed magnifications (Figure 1J–L).

### 3.6. Transcriptomic Analysis

**Figure 2:**
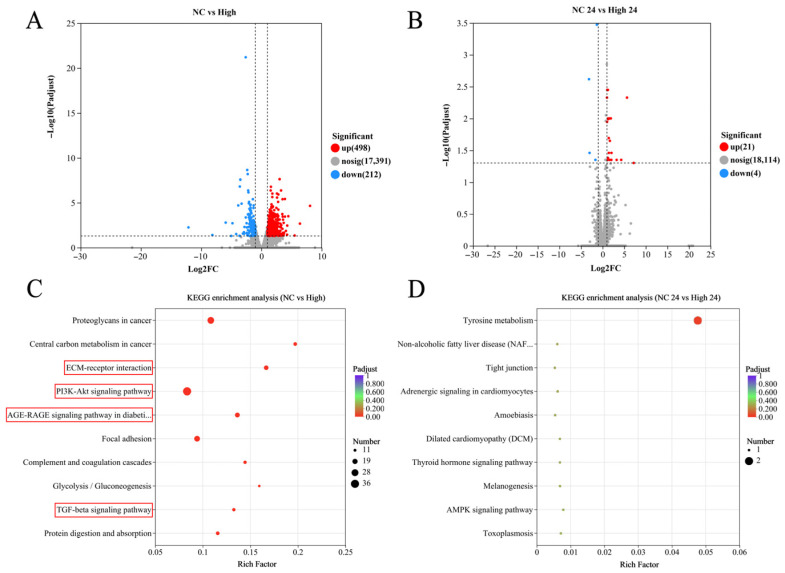
Comparison of skeletal muscle transcriptomic profiles between NC and High-treated Mongolian horses. (**A**,**B**) Volcano plots. (**C**,**D**) KEGG pathway enrichment. The pathways highlighted with red boxes indicate the key signaling pathways of interest discussed in this study. Due to layout constraints in the plotting software, truncated pathway names in the figure represent “AGE-RAGE signaling pathway in diabetic complications” in (**C**) and “Non-alcoholic fatty liver disease (NAFLD)” in (**D**).

**Figure 3:**
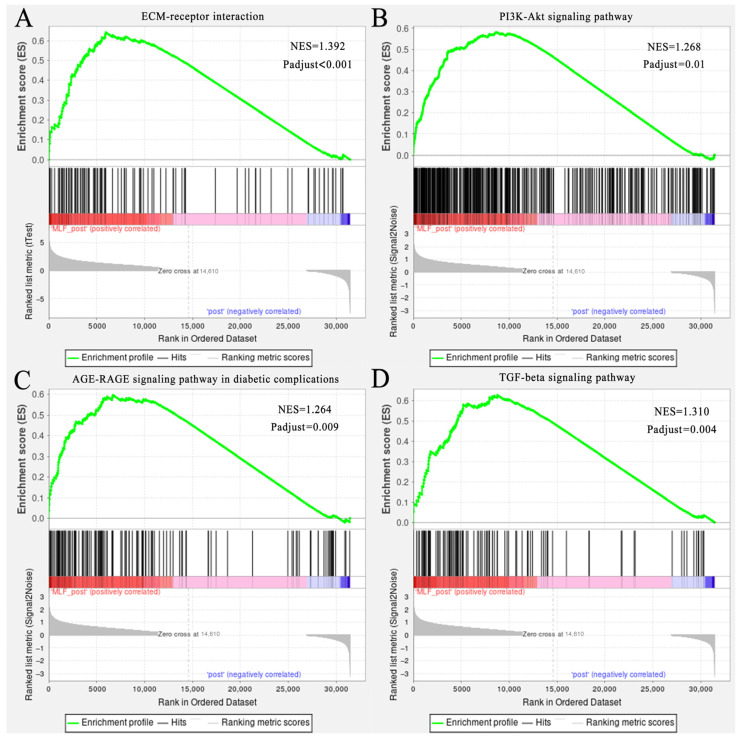
GSEA of key signaling pathways in skeletal muscle. (**A**) ECM–receptor interaction. (**B**) TGF-beta signaling pathway. (**C**) PI3K-Akt signaling pathway. (**D**) AGE-RAGE signaling pathway in diabetic complications.

To investigate the gene expression profiles in the gluteus medius following MLFs intervention, transcriptomic analysis was performed on Mongolian horses treated with 10 g/d MLFs. After 20 km exercise, 498 DEGs were upregulated and 212 DEGs were downregulated in the MLFs group compared to the NC group (Figure 2A). At 24 h post-exercise, 21 DEGs were upregulated and four DEGs were downregulated in the MLFs group (Figure 2B). Transcriptomic KEGG functional enrichment analysis revealed that after 20 km exercise, ECM–receptor interaction, PI3K-Akt signaling pathway, AGE-RAGE signaling pathway in diabetic complications, and TGF-beta signaling pathway were among the top nine enriched KEGG pathways (Figure 2C) (adjusted *p* < 0.05). Genes involved in these four signaling pathways exhibited significant coordinated changes in the skeletal muscle of MLF-treated horses. For instance, *TGFB1*, *TGFB3*, *TGFBR2*, and *THBS1* were significantly upregulated in the MLFs group (Figure S1A). Similar dynamic changes were observed in key genes involved in the PI3K-Akt signaling pathway (e.g., *IGF2*, *PPP2R2D*, and *PPP2R5*), ECM remodeling (e.g., *COL1A1*, *COL1A2*, *COL6A3*, and *COL14A1*), and AGE-RAGE signaling pathway in diabetic complications (e.g., *SELE*, *ICAM1*, *MAPK12*, and *JUN*) (Figure S1B–D). GSEA analysis further confirmed the changes in these four signaling pathways (Figure 3). Notably, transcriptomic data also revealed significant differences in the expression of core genes associated with skeletal muscle myogenesis and myofiber function. Compared with the 0 g/d MLFs group, the mRNA level of *MYOD* was significantly upregulated. Additionally, the mRNA levels of *MYOZ2*, *MYOM3*, *SOD3* and *GPX3* were significantly upregulated, whereas *MRF6* was significantly downregulated (Figure S1E–G). As shown in Figure S2, the expression trends at 0 h and 24 h post-exercise were highly consistent with the RNA-seq data, confirming the robustness of our transcriptomic results.

### 3.7. Proteomics Analysis

**Figure 4:**
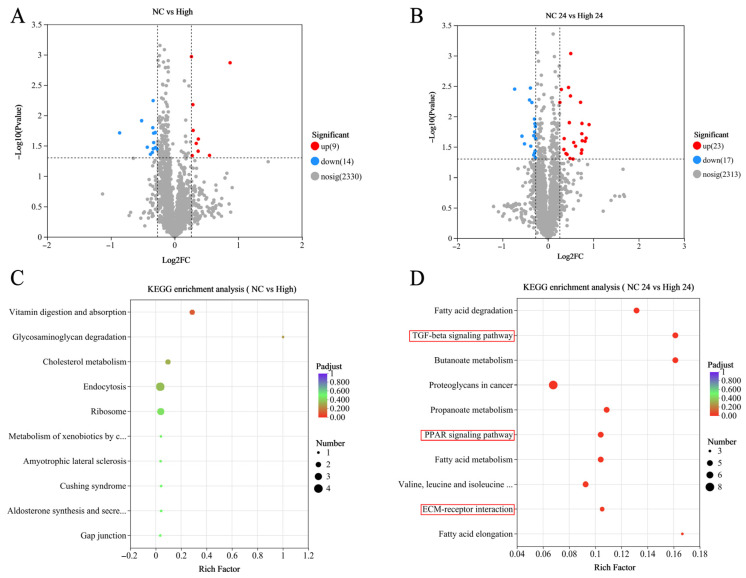
Comparison of skeletal muscle proteomic profiles between NC and High-treated Mongolian horses. (**A**,**B**) Volcano plots. (**C**,**D**) KEGG pathway enrichment. The pathways highlighted with red boxes indicate the key signaling pathways of interest discussed in this study. Due to layout constraints in the plotting software, truncated pathway names in the figure represent “Metabolism of xenobiotics by cytochrome P450” and “Aldosterone synthesis and secretion” in (**C**), and “Valine, leucine and isoleucine degradation” in (**D**).

**Figure 5:**
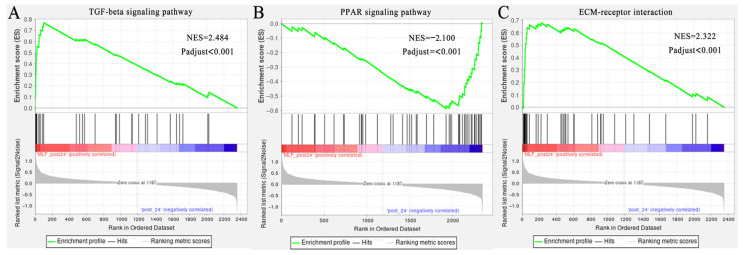
PSEA of key signaling pathways in skeletal muscle. (**A**) TGF-beta signaling pathway. (**B**) PPAR signaling pathway. (**C**) ECM–receptor interaction.

Furthermore, we analyzed the proteomic profiles of Mongolian horse muscle tissue based on the TMT-based quantitative proteomic approach. After 20 km exercise and at 24 h post-exercise, 25 and 40 DEPs were identified between the MLF-supplemented group and the NC group, respectively (Figure 4A,B). These proteins were subjected to visualized analysis via KEGG pathway enrichment (Figure 4D). Among these, the top 10 differential signaling pathways included the TGF-beta signaling pathway, PPAR signaling pathway, and ECM–receptor interaction (adjusted *p* < 0.05). Subsequent PSEA revealed that the expression levels of proteins associated with the ECM–receptor interaction and TGF-beta signaling pathway were higher in the MLFs group, whereas those associated with the PPAR signaling pathway were lower (Figure 5A,B). Consistent with these findings, targeted analysis at 24 h post-exercise demonstrated a significant upregulation of key proteins in the TGF-beta signaling pathway (e.g., COL6A3, COL6A6, COL14A1, and CFP) and ECM–receptor interaction networks (e.g., DCN, PRELP, LUM, and ASPN). Conversely, core proteins involved in the PPAR signaling pathway (such as ACADS, HADHA, ACAT1, and IVD) were significantly downregulated in the High-MLFs group (Figure S3).

## 4. Discussion

The aim of this study was to evaluate the protective effects of MLFs against exercise-induced muscle damage and to elucidate the underlying molecular mechanisms. The primary outcomes of this study reveal a robust correlation between the intervention with MLFs and the amelioration of blood biomarkers associated with oxidative stress, inflammation, and muscle damage in horses subjected to high-intensity exercise, suggesting the potential therapeutic significance of MLFs. Mongolian horses receiving MLFs exhibited significant changes in physiological parameters, including reduced immune cell counts, CK, and MDA levels. Notably, the optimal effect was observed at a dosage of 10 g. Indeed, numerous studies have shown the potential of MLFs, such as kaempferol [14], astragalin [26], and Mulberrin [27], in improving the antioxidant and anti-inflammatory status of animals.

The changes in hematological parameters induced by high-intensity exercise in horses in the present study are largely consistent with those previously described in human and equine studies [28,29]. High-intensity exercise led to increased levels of WBC, Lymph, Mon, and Gran in horses, consistent with reports by Johanna et al. [28] and Andriichuk et al. [30]. The increase in WBC is one of the common physiological responses to exercise, typically occurring after all types of physical activity [31]. The increase in WBC during exercise is biphasic, initially involving the mobilization of lymphocytes, followed by the mobilization of neutrophils and monocytes [32]. The exercise-induced leukocytosis is often likened to an inflammatory response, with the increase in WBC being proportional to the intensity of the immune system’s response to physiological stress [33,34]. Horses fed MLFs showed significant decrease in WBC, Mon, and Gran levels. Collectively, evidence in earlier studies and findings support that MLF can provide benefits in minimizing inflammatory response. Furthermore, the elevated RBC and HGB levels after the 20 km exercise potentially provide the physiological basis for enhanced oxygen transport, enabling better endurance and strength. However, further studies are needed to validate their functional effects on equine performance. These facilitative effects of MLFs are consistent with previous studies [14] indicating that MLFs possess anti-inflammatory and immunomodulatory effects, potentially aiding in the reduction in exercise-induced inflammatory responses.

Excessive exercise generates abundant ROS that can compromise the integrity of cellular membranes, leading to the leakage of numerous intracellular metabolites and proteins from muscle cells into the systemic circulation, particularly CK, LDH, and AST [35]. Horses supplemented with MLFs in the present study exhibited reductions in serum AST and CK levels. The reduction in CK levels is likely due to the antioxidant properties of MLFs, which can neutralize ROS produced during the electron transport chain in oxidative phosphorylation [36]. Another possibility for the decrease in CK levels is that MLFs could reduce the permeability of the vascular membrane, thereby reducing the flow of CK from inside cells to the bloodstream [37]. Although the serum CK levels in horses in this study remained within the normal reference range, an increase was observed after each exercise session. This observation is in line with expectations from the previous literature [38] and indicates that high-intensity exercise over 20 km is sufficient to cause muscle cell disturbance in this study. Considering that AST is a critical marker for liver health and CK for muscle damage in animals following acute exercise, the findings imply that MLFs exert a protective influence on both the liver and muscles.

The rise in ROS after high intensity exercise is thought to cause secondary damage to uninjured muscle fibers either by direct oxidative injury to biomolecules or by indirectly inducing inflammatory cytokines [39,40]. MDA is a well-recognized biomarker of oxidative stress, as it reflects the peroxidative damage caused by ROS to polyunsaturated fatty acids [41]. Meanwhile, in biological systems, CAT, SOD, and GPx are regarded as the three most potent antioxidant enzymes, acting as the primary defense against ROS [42]. Several studies have confirmed the increased lipid peroxidation during different forms of physical activity in sport horses [43,44,45]. Similarly, in the present study, MDA concentration increased significantly after physical activity, while a significant decrease after 24 h of rest was observed. It is noteworthy that during exercise and the subsequent 4 h recovery period dietary MLF supplementation significantly reduced the MDA concentration in Mongolian horses and increased serum T AOC, GPx and SOD activities. These results suggest that MLFs are associated with the mitigation of lipid peroxidation caused by exercise, aligning with findings from prior studies conducted on mouse models [46]. However, no statistically significant differences were observed in the antioxidant indices within the muscle tissue. This unforeseen result may be associated with the first-pass effect of MLFs in the liver [47], which reduces their bioavailability through ongoing metabolism and transport. Traditional view holds that flavonoids can directly scavenge excessive free radicals in the body. However, studies have shown that among the flavonoids taken orally, less than 5% can enter the blood circulation, and the remaining approximately 95% are fermented and decomposed by the intestinal flora [48]. Even so, the ability of flavonoids to alleviate EIMD lies in cell signaling stress response pathways, thereby enhancing the transcription of endogenous antioxidant enzymes [49,50]. In line with this mechanism, our data revealed an upregulation of *GPX3* and *SOD3* mRNA levels following MLF supplementation, pointing toward a potential activation of endogenous antioxidant defenses against exercise-induced oxidative stress at the transcriptional level.

Primary muscle injury is characterized by progressive degradation of muscle fibers, disruption of sarcomeric architecture, Z-line streaming, and loss of essential cytoskeletal proteins [51]. In our experiment, horses subjected to a 20 km exercise exhibited clear muscle fiber damage; although some recovery of myofibrillar structure was observed after a 24 h recovery period, the lack of distinct I Band and the tortuosity of the Z-line suggest that complete recovery of myofibrillar structure after intense exercise may require more than 24 h. In the MLFs treatment, horses showed better recovery states immediately post-exercise and at the 24 h mark. Further transcriptomic data indicate that pretreatment with MLFs significantly upregulates the mRNA expression of *MYOZ2* and *MYOM3* in horse skeletal muscle. Previous studies have shown that the *MYOZ2* and *MYOM3* genes, by encoding core structural proteins of the Z-line and M-line in muscle fibers respectively, play an important role in preserving muscle structural integrity [52,53]. Myogenesis is the principal repair mechanism after muscle injury, governed by differential expression of myogenic regulatory factors (MRFs); MyoD promotes satellite cell activation toward the myogenic lineage, whereas MRF6 mainly controls terminal differentiation of myoblasts [54,55]. In this study, the mRNA expression of *MYOD1* was significantly upregulated, whereas that of *MRF6* was significantly downregulated, indicating that MLFs may accelerate the regeneration and repair of injured muscle fibers by promoting early satellite cell activation and proliferation. Concordantly, this is consistent with our observations in myofibrillar structure. Through KEGG enrichment analysis of skeletal muscle samples from horses collected immediately post-exercise and 24 h later, it is suggested that the PI3K/AKT pathway, TGF-β pathway, and ECM–receptor interaction, as the most significantly co-enriched pathways at both post-exercise time points, play important roles in MLF-mediated mitigation of exercise-induced muscle injury in horses.

The core of myofiber injury lies in an imbalance between protein synthesis and degradation (i.e., synthesis rate < degradation rate) [56]. Reported evidence indicates that skeletal muscle protein synthesis and degradation are regulated by the mechanistic target of rapamycin (mTOR) pathway and the ubiquitin–proteasome system [57]. The PI3K/Akt pathway serves as a key upstream axis that activates both the mTOR and FOXO signaling cascades [58]. Concurrently, the TGF-β pathway positively regulates muscle protein synthesis [59]. Among these, bone morphogenetic proteins, members of the TGF-β superfamily, can promote skeletal–muscle hypertrophy by activating the mTOR pathway and the SMAD1/5/8 cascade [60]. Evidence shows that mulberry leaf flavonoids activate PI3K/AKT/mTOR and TGF-β pathways, thereby enhancing skeletal–muscle development in broiler chickens [61]. Similarly, in mouse models, active constituents of mulberry leaves activate the TGF-β/Smad pathway, alleviating oxidative-stress-induced renal fibrosis [62]. Moreover, TGF-β (particularly TGFB1) is among the earliest cytokines upregulated after tissue injury, regulating ECM synthesis, remodeling, and degradation [63,64]. During muscle repair, TGF-β activates transcription of genes encoding core ECM proteins, such as COL1A1, COL1A2 (type I collagen subunits) and FN1 (fibronectin), thereby furnishing a structural scaffold for myofiber regeneration [65]. Collectively, these findings imply that MLFs may mitigate exercise-induced muscle injury in horses by modulating these specific pathways to favor protein synthesis and structural recovery. While our omics data highlight potential pathways, future functional studies are necessary to confirm the precise signaling mechanisms underlying the protective effects of MLFs.

## 5. Conclusions

In summary, this study supports that MLFs play an important role in the process of mitigating high-intensity exercise-induced muscle damage in Mongolian horses. The comprehensive hemotological and cytological data indicate that the processes involved include attenuation of inflammatory responses, correction of metabolic disturbances, regulation of protein metabolic balance, upregulation of structural proteins, activation of antioxidant defenses, and promotion of the proliferation and differentiation of skeletal muscle satellite cells. Therefore, MLF supplementation may represent an effective phytotherapy for reducing oxidative stress and muscle damage induced by strenuous exercise. Within the tested range, the 10 g/d dose provided more effective protection than the 5 g/d dose.

## Figures and Tables

**Table 1:** Effects of dietary MLF supplementation on hematological indices of horses at different exercise distances.

Item	Dosage (g/d)	0 km	5 km	20 km	Pooled SEM	*P*_D_	*P*_M_	*P*_D×M_
WBC (10^9^/L)	0	11.08	12.4	13.23	0.294	<0.001	0.001	0.864
	5	10.25	11.68	12.78				
	10	9.7	10.3	11.2				
Lymph (10^9^/L)	0	3.95	4.87	5.4	0.171	<0.001	0.09	0.924
	5	3.57	4.77	5.35				
	10	3.37	4.24	5.2				
Mon (10^9^/L)	0	0.6	0.7	0.81	0.018	<0.001	<0.001	0.651
	5	0.56	0.62	0.72				
	10	0.5	0.55	0.62				
Gran (10^9^/L)	0	5.98	6.3	7.02	0.211	<0.001	0.046	0.684
	5	5.43	6.15	6.83				
	10	4.98	5.98	6.69				
RBC (10^12^/L)	0	6.77	7.65	7.08	0.197	0.003	0.002	0.997
	5	7.77	8.49	8.02				
	10	7.13	8.08	7.48				
HGB (g/L)	0	95.1	108	98.8	2.210	<0.001	<0.001	0.99
	5	102	116.29	109.14				
	10	100.86	118.5	109				

*P*_D_, *P*_M_, and *P*_D×M_ represent the main effects of exercise distance, MLFs dosage, and their interaction, respectively.

**Table 2:** Effects of dietary MLF supplementation on hematological indices of horses during the post-exercise recovery period.

Item	Dosage (g/d)	0 h Post	1 h Post	4 h Post	Pooled SEM	*P*_T_	*P*_M_	*P*_T×M_
WBC (10^9^/L)	0	13.23	12.2	11.58	0.285	0.001	0.001	0.875
	5	12.78	11.68	10.58				
	10	11.2	10.9	9.8				
Lymph (10^9^/L)	0	5.4	4.4	3.85	0.150	<0.001	0.426	0.999
	5	5.35	4.33	3.73				
	10	5.2	4.13	3.5				
Mon (10^9^/L)	0	0.81	0.7	0.62	0.018	<0.001	<0.001	0.677
	5	0.72	0.64	0.58				
	10	0.62	0.56	0.53				
Gran (10^9^/L)	0	7.02	6.7	6.25	0.131	<0.001	0.024	0.595
	5	6.83	6.4	6.1				
	10	6.69	6.3	5.38				
RBC (10^12^/L)	0	7.08	6.38	6.95	0.201	0.278	0.001	0.758
	5	8.02	7.72	7.7				
	10	7.48	7.29	7.21				
HGB (g/L)	0	98.8	93.3	95.8	2.213	0.14	<0.001	0.938
	5	109.14	104.71	102				
	10	109	103.38	106.08				

*P*_T_, *P*_M_, and *P*_T×M_ represent the main effects of post-exercise recovery time, MLFs dosage, and their interaction, respectively.

**Table 3:** Effects of dietary MLF supplementation on biochemical parameters of horses at different exercise distances.

Item	Dosage (g/d)	0 km	5 km	20 km	Pooled SEM	*P*_D_	*P*_M_	*P*_D×M_
LDH (U/L)	0	262.4	262.92	285.64	6.553	0.008	0.041	0.983
	5	263.33	255.67	282.33				
	10	248.27	238.1	267.78				
ALP (U/L)	0	191.24	184	189.61	2.971	0.006	<0.001	0.517
	5	225.66	214.72	225.94				
	10	220.48	198.14	207.6				
AST (U/L)	0	267.83	280.53	294.1	6.243	<0.001	<0.001	0.565
	5	228.43	222.4	258.83				
	10	204.43	218.97	253.93				
UREA (mmol/L)	0	6.96	7.88	8.28	7.708	<0.001	0.003	0.852
	5	6.74	7.62	7.84				
	10	6.52	7.44	7.46				
CREA (mmol/L)	0	86.18	92.32	106.42	1.812	<0.001	0.012	0.997
	5	83.43	90.43	104.08				
	10	78.93	85.05	97.42				
TC (mmol/L)	0	1.61	1.69	1.82	0.021	<0.001	0.001	0.799
	5	1.58	1.64	1.74				
	10	1.52	1.57	1.65				
TG (mmol/L)	0	0.24	0.26	0.34	0.014	0.002	0.039	0.754
	5	0.22	0.23	0.28				
	10	0.21	0.23	0.26				
GLU (mmol/L)	0	3.55	4.65	5.17	0.137	<0.001	0.142	0.3
	5	3.87	4.38	5.26				
	10	3.82	5.2	5.34				
CK (mmol/L)	0	176.63	195.18	221.55	4.888	<0.001	<0.001	0.953
	5	150.7	180.08	193.68				
	10	144.15	167.63	186.65				

*P*_D_, *P*_M_, and *P*_D×M_ represent the main effects of exercise distance, MLFs dosage, and their interaction, respectively.

**Table 4:** Effects of dietary MLF supplementation on biochemical parameters of horses during the post-exercise recovery period.

Item	Dosage (g/d)	0 h Post	1 h Post	4 h Post	Pooled SEM	*P*_T_	*P*_M_	*P*_T×M_
LDH (U/L)	0	285.64	285.05	278.54	6.828	0.978	0.056	0.892
	5	282.33	288.9	289.8				
	10	267.78	262.92	272.54				
ALP (U/L)	0	189.61	188.61	205.46	3.295	0.001	<0.001	0.265
	5	225.94	224.3	232				
	10	207.6	221.58	238.28				
AST(U/L)	0	294.1	282.06	276.6	6.033	0.002	<0.001	0.751
	5	258.83	222.73	226.53				
	10	253.93	223.8	220.55				
UREA (mmol/L)	0	8.28	8.03	7.14	0.104	<0.001	0.004	0.372
	5	7.84	7.78	7.21				
	10	7.46	7.38	7				
CREA (mmol/L)	0	106.42	95.12	89.2	1.776	<0.001	0.004	0.97
	5	104.08	91.13	84.9				
	10	97.42	88.05	78.88				
TC (mmol/L)	0	1.82	1.73	1.63	0.021	<0.001	<0.001	0.838
	5	1.74	1.65	1.56				
	10	1.65	1.54	1.52				
TG (mmol/L)	0	0.34	0.19	0.15	0.018	<0.001	0.322	0.902
	5	0.28	0.18	0.14				
	10	0.26	0.13	0.13				
GLU (mmol/L)	0	5.17	4.1	3.83	0.148	<0.001	0.456	0.425
	5	5.26	3.87	3.86				
	10	5.34	4.48	3.78				
CK (mmol/L)	0	221.55	201.85	184.03	4.804	<0.001	<0.001	0.977
	5	193.68	183.98	160.13				
	10	186.65	176.13	153.7				

*P*_T_, *P*_M_, and *P*_T×M_ represent the main effects of post-exercise recovery time, MLFs dosage, and their interaction, respectively.

**Table 5:** Effects of dietary MLF supplementation on serum antioxidant indices of horses at different exercise distances.

Item	Dosage (g/d)	0 km	5 km	20 km	Pooled SEM	*P*_D_	*P*_M_	*P*_D×M_
T-AOC (mM)	0	0.47	0.43	0.34	0.014	<0.001	<0.005	0.814
	5	0.49	0.46	0.41				
	10	0.52	0.49	0.44				
SOD (U/mL)	0	16.68	14.99	13.44	0.403	<0.001	0.001	0.964
	5	18.85	16.77	14.66				
	10	19.35	17.1	15.58				
CAT (U/mL)	0	11.36	11.28	10.42	0.244	<0.004	0.623	0.991
	5	11.65	11.43	10.56				
	10	11.83	11.39	10.67				
GPx (U/mL)	0	356.67	345.8	313.97	4.721	<0.001	<0.001	0.815
	5	381.74	359.84	336.06				
	10	393.78	369.12	355.9				
MDA (nmol/mL)	0	7.36	8.32	9.07	0.353	<0.007	<0.001	0.979
	5	6.48	7.07	8.15				
	10	5.67	6.22	6.89				

*P*_D_, *P*_M_, and *P*_D×M_ represent the main effects of exercise distance, MLFs dosage, and their interaction, respectively.

**Table 6:** Effects of dietary MLF supplementation on serum antioxidant indices of horses during the post-exercise recovery period.

Item	Dosage (g/d)	0 h Post	1 h Post	4 h Post	Pooled SEM	*P*_T_	*P*_M_	*P*_T×M_
T-AOC (mM)	0	0.34	0.38	0.43	0.012	0.026	<0.001	0.105
	5	0.41	0.44	0.47				
	10	0.44	0.47	0.51				
SOD (U/mL)	0	13.44	15.26	16.22	0.374	<0.001	<0.001	0.595
	5	14.66	15.83	18.64				
	10	15.58	18.03	19.38				
CAT (U/mL)	0	10.42	10.59	10.75	0.246	0.131	0.179	0.936
	5	10.56	10.88	11.31				
	10	10.67	11.27	11.65				
GPx (U/mL)	0	313.97	338.03	352.17	4.726	<0.001	<0.001	0.771
	5	336.06	351.76	371.33				
	10	355.9	363.9	397.91				
MDA (nmol/mL)	0	9.07	8.29	7.59	0.262	0.001	<0.001	0.5
	5	8.15	8.14	6.07				
	10	6.89	6.66	5.86				

*P*_T_, *P*_M_, and *P*_T×M_ represent the main effects of post-exercise recovery time, MLFs dosage, and their interaction, respectively.

**Table 7:** Effects of dietary MLF supplementation on muscle antioxidant indices of horses during the post-exercise recovery period.

Item	Dosage (g/d)	0 h Post	24 h Post	Pooled SEM	*P*_T_	*P*_M_	*P*_T×M_
T-AOC (mM)	0	0.15	0.34	0.061	0.02	0.512	0.61
	10	0.16	0.45				
SOD (U/mL)	0	31.64	42.11	5.217	0.194	0.256	0.954
	10	40.72	50.36				
CAT (U/mL)	0	20.74	32.21	2.773	0.373	0.447	0.618
	10	30.87	34.57				
GPx (U/mL)	0	762.6	794.87	88.801	0.666	0.559	0.862
	10	814.31	889.44				
MDA (nmol/mL)	0	6.53	4.3	0.920	0.113	0.358	0.937
	10	5.26	3.22				

*P*_T_, *P*_M_, and *P*_T×M_ represent the main effects of post-exercise recovery time, MLFs dosage, and their interaction, respectively.

## Data Availability

All data generated or analyzed during this study are included in this published article.

## Supplementary Materials

The following supporting information can be downloaded at: https://www.mdpi.com/article/10.3390/ani16101548/s1, Figure S1: Expression profiles of key DEGs identified from transcriptomic analysis at 0 h post-exercise. Bar charts illustrating the mRNA expression levels of representative DEGs involved in the (A) TGF-beta signaling pathway, (B) PI3K-Akt signaling pathway, (C) ECM–receptor interaction, (D) AGE-RAGE signaling pathway, (E) Myogenic regulatory factors, (F) Sarcomeric protein genes, and (G) Antioxidant enzyme genes. Data are presented as mean ± SD. * *p* < 0.05, ** *p* < 0.01, *** *p* < 0.001; Figure S2: Comparison of RNA Seq and RT-q PCR results in both NC vs. High (A) and NC 24 vs. High 24 comparison groups (B). Figure S3. Expression profiles of key DEPs involved in core signaling pathways at 24 h post-exercise. Bar charts illustrating the relative expression levels of representative proteins associated with the (A) TGF-beta signaling pathway, (B) ECM–receptor interaction, and (C) PPAR signaling pathway in the skeletal muscle of the NC and High-MLFs groups. Data are presented as mean ± SD. * *p* < 0.05, ** *p* < 0.01, *** *p* < 0.001; Table S1. Groups by Latin square test method; Table S2. Effects of dietary MLF supplementation on average heart rate and average speed of horses during exercise; Table S3. Primers sequence.

## Abbreviations

The following abbreviations are used in this manuscript:

NC	Normal Control
Low	Low-Dose MLFs Treatment
MLFs	Mulberry leaf flavonoids
EIMD	Exercise-induced muscle damage
ROS	Reactive oxygen species
WBC	White blood cells
Lymph	Lymphocyte count
Mon	Monocyte count
Gran	Granulocyte count
RBC	Red blood cell count
HGB	Hemoglobin
AST	Aspartate aminotransferase
CREA	Creatinine
LDH	Lactate dehydrogenase
ALP	Alkaline phosphatase
TC	Total Cholesterol
TG	Triglycerides
GLU	Glucose
CK	Creatine Kinase
GPx	Glutathione peroxidase
SOD	Superoxide dismutase
CAT	Catalase
T-AOC	Total antioxidant capacity
MDA	Malondialdehyde
TEM	Transmission electron microscope
DEGs	Differentially expressed genes
DEPs	Differentially expressed proteins
GSEA	Gene Set Enrichment Analysis
PSEA	Protein Set Enrichment Analysis
LMM	Linear Mixed Model
SEM	Standard Error of the Mean
