# Supplementary material for: Transcriptomic and Proteomic Analysis of the Skeletal Muscle Revealed the Effects and Mechanism of Mulberry Leaf Flavonoids on Alleviating Exercise-Induced Muscle Damage in Mongolian Horses
Source: Animals (Basel). 2026 May 18;16(10):1548. doi: 10.3390/ani16101548 (PMC13203402; doi:10.3390/ani16101548)
Supplement: Supplementary file 1 [file animals-16-01548-s001.zip › animals-4276665-supplementary.pdf]

Supplementary Table S1. Groups by Latin square test method

Item	Group 1 (n=4)	Group 2 (n=4)	Group 3 (n=4)
Phase 1	NC (0 g/d)	Low (5 g/d)	High (10 g/d)
Phase 2	Low (5 g/d)	High (10 g/d)	NC (0 g/d)
Phase 3	High (10 g/d)	NC (0 g/d)	Low (5 g/d)

Supplementary Table S2. Effects of dietary MLFs supplementation on average heart rate and average speed of horses during exercise.

Item	0 g/d	5 g/d	10 g/d	SEM	*P*-value
average heart rate (bpm)	120.88	124	121.79	2.550	0.895
average speed (km/h)	14.44	14.91	14.56	0.318	0.846

Supplementary Table S3. Primers sequence

Gene	Primer Sequence (5′- 3′)
ISLR	F: TGTGGCATCGTGTGGTTCAAGAC<br>R: GGCAGCAGGCGGTTCAGC
DACT2	F: AAGTCCACTCCTCTCCTCTGC<br>R: TACACCTCCTTGCCACCCTTG
GBP6	F: TGGGAATCGGCTGAGGACTCTG<br>R: CTGTAATGGTCCGCTGCCTTCTG
MAPK12	F: CGCCTCCCGCAGACTTTGTG<br>R: GCACCAGCATTCTCTCCAGCAG
TGM2	F: GCGTGAGCCAGAGCATGAGC<br>R: GGCGGCAAGCGTGGTCTTC
CHAC1	F: CAGTGCTTGGCGGCTATGATACC<br>R: GATCTGTGTAGCAATGGCCTCCTC
PFKFB3	F: GCTGGAGCGGCAGGAGAAC

	R: TGGAGCGGGCATTTCAGGTAAG
SLC7A8	F: AACCTTCCCAGAGCCATCTTCATC<br>R: ATTGCGGTGACATAAGCGATATTGG
DDI2	F: TTCCTGGCGTGTTCCTTCTCTATAC<br>R: GGTGGTCTGCGAGCCTGTG
CREG1	F: GCAGGAACTGTTACCAAGGTGAATG<br>R: CGAAGAACCAATTATGGCTGGAAGG
H1-3	F: CCAAGAAGGCGGCTAAGAGTCC<br>R: GTAACCTTCGGCTTGCCAGATTTC
DHCR24	F: CTCTTCCTGCTGCCGCTATCG<br>R: CTGCCCTGCTCCTTCCATTCC
TTC38	F: GCCGCCTTGACCATTTACGATAAC<br>R: CACCGCTCACCCACAGACAC
RASSF8	F: GGACAAGCCACCAAACGATTACAG<br>R: ACTGCTGGAGGTTGACTTGCC
ACTC1	F: CGCTGTCTTCCCGTCCATCG<br>R: TCGCTTGCTCTGTGCCTCATC
LAMC3	F: CTACTATGCCGTGTCTGACTTCTCC<br>R: TCTGTGCCCGTGGTGTTGTG
MSS51	F: ACTTGGAAGAACGAGCAGGAAGG<br>R: GGAGTCACTGATGAGGGAGGTTTC

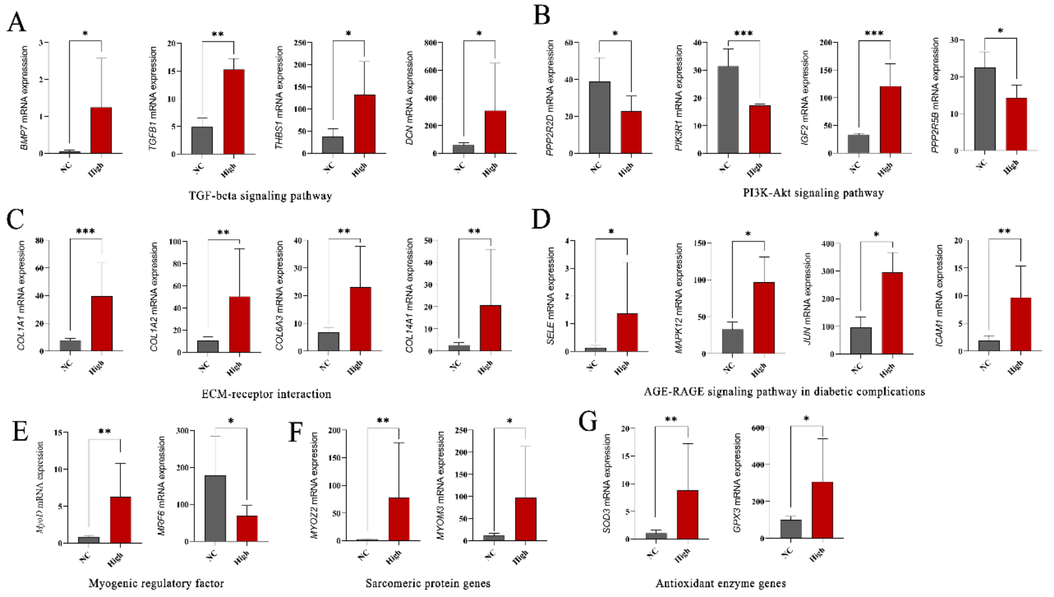

Supplementary Figure S1. Expression profiles of key DEGs identified from transcriptomic analysis at 0 h post-exercise. Bar charts illustrating the mRNA expression levels of representative DEGs involved in the (A) TGF-beta signaling pathway, (B) PI3K-Akt signaling pathway, (C) ECM-receptor interaction, (D) AGE-RAGE signaling pathway, (E) Myogenic regulatory factors, (F) Sarcomeric protein genes, and (G) Antioxidant enzyme genes. Data are presented as mean ± SD. *$P < 0.05$, **$P < 0.01$, ***$P < 0.001$.

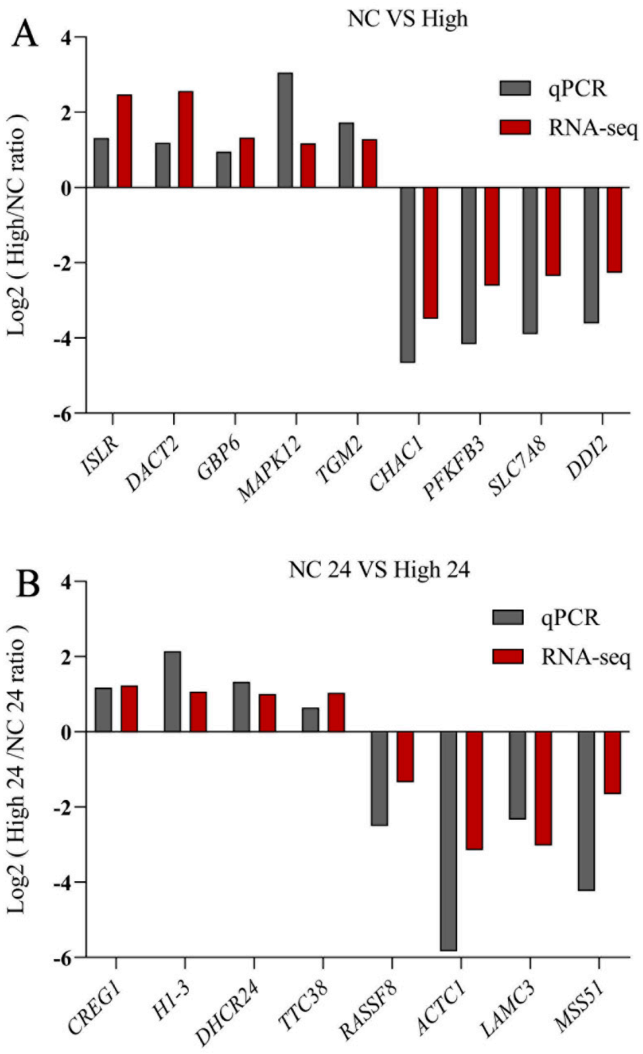

Supplementary Figure S2. Comparison of RNA Seq and RT-q PCR results in both NC vs High (A) and NC 24 vs High 24 comparison groups (B).

.

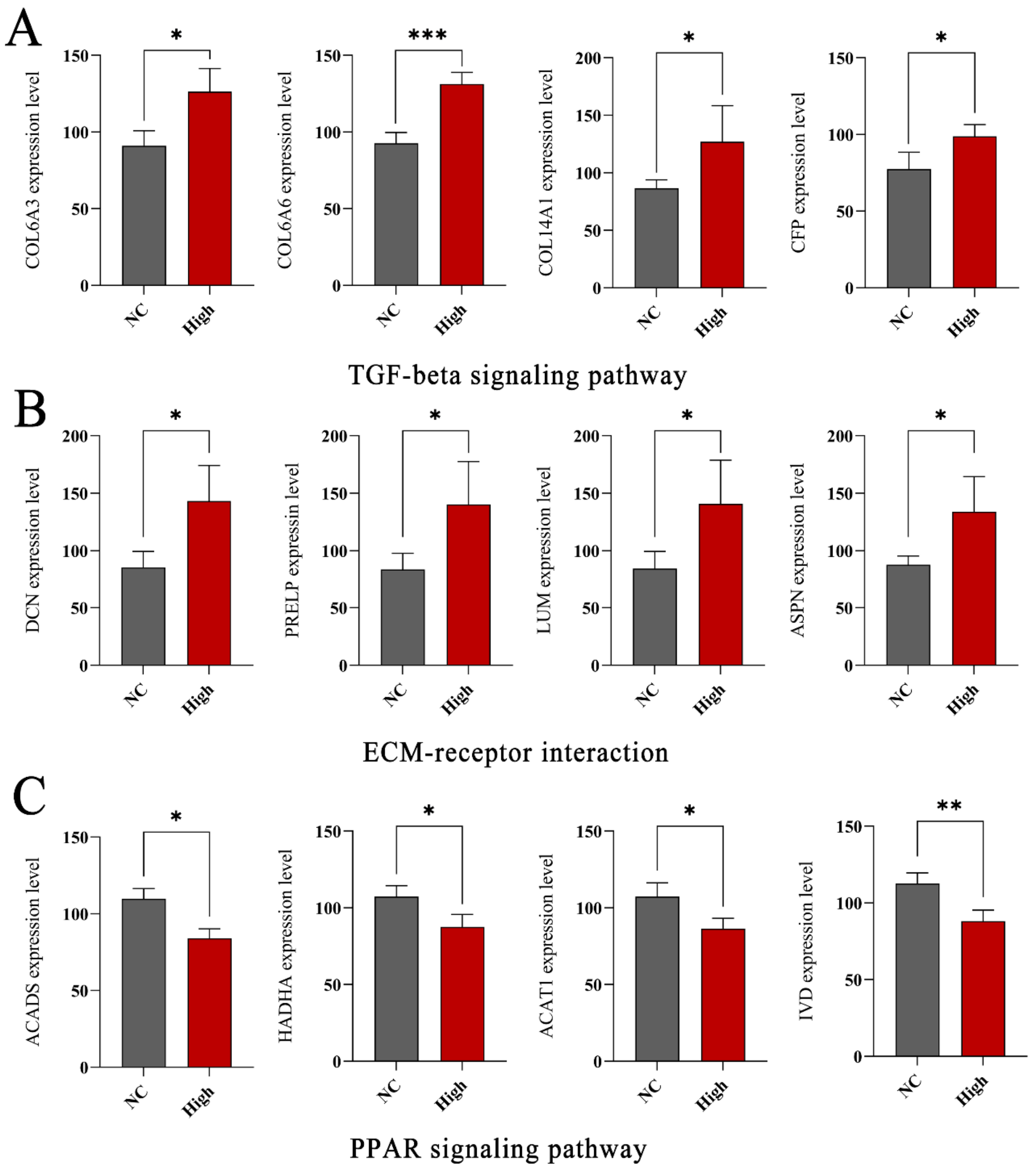

Supplementary Figure S3. Expression profiles of key DEPs involved in core signaling pathways at 24 h post-exercise. Bar charts illustrating the relative expression levels of representative proteins associated with the (A) TGF-beta signaling pathway, (B) ECM-receptor interaction, and (C) PPAR signaling pathway in the skeletal muscle of the NC and High MLFs groups. Data are presented as mean ± SD. *$P < 0.05$, **$P < 0.01$, ***$P < 0.001$.
